# Rapid and Deep versus Normal Breathing in Salbutamol Inhalation Effectiveness; a Letter to Editor

**DOI:** 10.22037/aaem.v9i1.1122

**Published:** 2021-05-26

**Authors:** Faeze Zeinali, Naser Mohammad Karimi, Mohamadali Jafari, Ebrahim Akbarzadeh Moghadam

**Affiliations:** 1 Emergency Medicine Department, Shahid Sadoughi Hospital ,Shahid sadoughi University of Medical Sciences, Yazd, Iran.

**Keywords:** Inhalation spacers, asthma, dyspnea, pulmonary disease, chronic obstructive, peak expiratory flow rate

**Dear Editor;**

Metered dose Inhalers (MDIs) are widely used in the management of patients with asthma and choronoc obstractyive polmunary disease (COPD). Studies comparing the efficacy of inhalers versus nebulizers have shown no significant difference (1, 2). Good inhaler technique is essential to improve patient compliance and control of symptom, and diminish side effects. The usual technique is to use 5 tidal breaths. The Global Initiative for Asthma guidelines suggest that patients can take a slow and single breath to inhale the drug or do tidal breathing. The total lung deposition of an inhaled treatment is strongly affected by the speed of inhalation. For ideal drug delivery, it is vital that the inspiratory flow at the beginig of inhalation is fast enough to break up the formulation of the metered dose to yield particles of a size distribution that will enter the peripheral airways (3-5). Failure to attain this high internal force increases the likelihood of the dose affecting the oropharynx. Errors in inhaler technique are linked with lower drug deposition to the lungs and poor clinical control, and may lead to increased emergency ward admissions and higher treatment costs (6, 7). The authors of this letter compared the effectiveness of two inhalation methods of salbutamol spray (rapid and deep breathing vesrus normal breathing with tital volume) in management of patients with respiratoty distrease in emergency department. This randomized clinical trial was conducted on 14 to 75 year-old patients who visited the emergency department of Shahid Sadoughi Hospitals, Yazd, Iran, with asthma exacerbation. The patients were randomly divided into two inhalation technique groups using simple random sampling. One group received 200μg salbutamol (using salbutamol MDI) via rapid and deep breathing and another group received the same amount through 5 normal tidal breaths**. **All patients gave informed consent and the protocol of the investigation was approved by the local ethics committee (Ethics code:IR.SSU.MEDICINE.REC.1395.181). This trial was registred in Iranian registry of clinical trials (IRCT20171531038154N1). Peak expiratory flow (PEF) was determined before and 5, 10, and 15 minutes after inhaling 200μg Salbutamol in both groups. 

110 patients with the mean age of 39.5 ±16.7 years were randomly assigned to two groups (52% males). The two groups were similar regarding gender (p 0.088), age (p = 0.083), and mean baseline PEF (p = 0.75). Mean PEF rates of the two groups at baseline and 5, 10 and 15 minutes after salbutamol administration are presented in table 1. 

Significant improvment in PEF, from baseline to after intervention, was observed in both groups (p < 0.001). In addition, a significant improvement was observed in PEF, from 5 minutes to 10 and 15 minutes after treatment in both groups (p < 0.001). However, PEF was not significantly different between groups 5 (p = 0.56), 10 (p = 0.18), and 15 (p = 0.10) minutes after treatment. 

Boskabady’s study showed that giving proper technique instruction to asthmatic patients could improve bronchodilator responses, such as an increase in PEF (8). Rahmati et al. showed that proper use of MDI, with or without spacer, could increase PEF in asthmatic patients (9). Patients can take a slow single inhalation instead of tidal breathing (10). Stephen et al. did a randomized controlled trial to exibit that there was no clinically significant difference between PEF with one maximum dose inhalation and then breath-holding and 5 tidal breaths at the time of salbutamol inhalation using MDI in 82 asthmatic children 5–15 years of age (11). Another study by Schultz et al. evaluated the number of inhalations required to inhale salbutamol from various spacers/valved holding chambers. They concluded that one maximal dose inhalation (without breath-hold) did not improve delivery of drug in comparison with tidal breathing, which is in compliance with the discovery of our study (12). A similar study demonstrated that bronchodilators delivered by MDI via nebuhaler have comparable outcome when given by six tidal breaths or the more difficult two maximum breaths plus breath-hold (13). 

Inhaler technique assessment is an elemental part of the ordinary treatment of anybody suffering from asthma or COPD. For inhaled medicines such as salbutamol, additional attention to the application technique is needed to select the best dosage, but there are very few studies on this subject to estimate the effectiveness of each method. Previously, studies have shown that a considerable portion of patients do not operate/use their inhaler devices correctly (7, 14-16), this may contribute to reduction of medication delivery and poor disease control (17-20). In this regard, the most frequent error observed with using MDI was the step of waiting for 30 seconds between inhalations. Lack of proper exhalation prior to inhalation was reported in 25% of the inhaler users (6). Educational aids could be effective in this process (21), but many patients may use their devices incorrectly even after training. Our study suggests that rapid and deep inhalation without a breath-hold technique is not better than 5 tidal breaths technique in correcting PEF in those suffering from asthma. Therefore, patients should be given consultations for choosing the most appropriate technique for them when using inhaled medications. The data of this study may not be generalizable, because it was a single-hospital-based study. Another limitation of this study was that we did not assess the side effects of the two methods.

**Table 1:** Comparing the peak of expiratory flow (PEF) between the two groups at baseline and different times after treatment

**Time**	**Groups (breathinhg method)**		**P value**
	**Normal**	**Rapid and deep**	
**Baseline**	266.0 ± 81.7	261.0 ± 98.0	0.75
**5 minutes**	302.0 ± 86.74	292.0 ± 104.0	0.56
**10 minutes**	331.0 ± 92.1	304.0 ± 107.0	0.18
**15 minutes**	342.0 ± 100.0	309.0 ± 109.0	0.10

Data are presented as mean ± standard deviation.

## 1. Declarations

### 1.1. Acknowledgement

The authors would like to thank Dr. S.Mahmood Hosseini and Miss Amineh Brumand for their valuable comments on English writing of the manuscript.

### 1.2. Author contributions

Naser Mohamad karimi designed the study, collected the data, and performed analysis. Faeze Zeinali.N performed the analysis, wrote the draft and submitted the manuscript. Both athours read and confirmed the final version of the manuscript before submission.

### 1.3. Conflict of interest

There was no financial payment to the authors for writing this manuscript, and there is no conflict of interest about this article.

### 1.4. Funding support

None.
